# Intraoperative nerve monitoring reduces recurrent laryngeal nerve palsy in endoscopic thyroid surgery: a trial sequential analysis

**DOI:** 10.1097/JS9.0000000000001411

**Published:** 2024-03-27

**Authors:** Kuo-Chuan Hung, Wei-Ting Wang, I-Wen Chen

**Affiliations:** a Department of Anesthesiology, Chi Mei Medical Center; b Department of Anesthesiology, Chi Mei Medical Center, Liouying, Tainan City; c Department of Anesthesiology, E-Da Hospital, I-Shou University, Kaohsiung City, Taiwan

*Dear Editor,*

We read with great interest the article by Liu *et al*.^1^ titled ‘Effectiveness of recurrent laryngeal nerve monitoring during endoscopic thyroid surgery: systematic review and meta-analysis’. The authors performed a comprehensive meta-analysis to evaluate the effectiveness of intraoperative nerve monitoring (IONM) in endoscopic thyroid surgery. They found that the use of IONM significantly reduced the incidence of transient recurrent laryngeal nerve (RLN) palsy from 6.15% to 2.64%, total RLN palsy from 6.90% to 2.83%, and a relative risk reduction of 59%^1^. However, IONM did not significantly reduce the incidence of permanent RLN palsies. The authors also found that IONM reduced RLN location time and increased the recognition rate of the superior laryngeal nerve. Based on these findings, they recommend the use of IONM for thyroid malignancies^1^. The findings of this meta-analysis are important, as they provide evidence for the beneficial role of IONM in reducing RLN injury during endoscopic thyroid surgery. RLN palsy is a serious complication that can significantly affect patients’ quality of life. Therefore, interventions to minimize these complications are clinically relevant. We congratulate the authors for conducting this well-designed study.

Although the authors’ recommendation to use IONM for malignant thyroid tumors is reasonable based on their analysis^1^, the evidence should be verified further before widely implementing IONM in clinical practice. One way to assess the conclusiveness of meta-analysis findings is through trial sequential analysis (TSA)^2,3^. TSA accounts for sequential testing of significance each time a new trial is added, adjusting thresholds for statistical significance to control for the risk of false-positive results^4^. To evaluate the efficacy of IONM for total RLN palsy, we performed TSA (TSA viewer version 0.9.5.10 Beta) using raw data from the study by Liu *et al*.^1^. We entered these parameters: a 59% relative risk reduction (as observed in the initial meta-analysis^1^), a control group incidence of 6.9%, an alpha level of 5%, and a beta level of 80%. TSA results showed that the cumulative *z*-curve crossed the required information size boundary (Fig. 1) indicating that the evidence was sufficient and conclusive.

**Figure 1:**
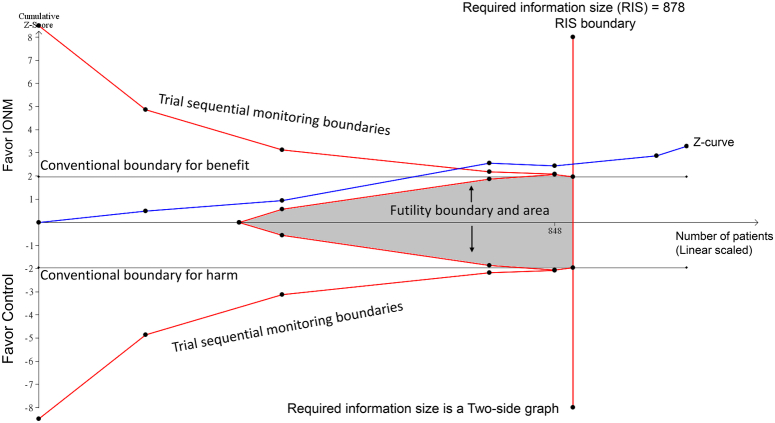
Trial sequential analysis evaluating the effect of intraoperative nerve monitoring (IONM) on reducing total recurrent laryngeal nerve palsy in endoscopic thyroid surgery. The required information size of 878 patients was calculated based on a relative risk reduction of 59%, an incidence in the control group of 6.9%, *α*=5%, and *β*=80%. The cumulative *z*-curve (blue line) crossed the required information size boundary (vertical red lines), indicating sufficient evidence to support 59% risk reduction with IONM.

In conclusion, the TSA results support that IONM reduces the risk of total RLN palsy by 59% in endoscopic thyroid surgery, strengthening the confidence in the meta-analysis findings. Further studies are still warranted to evaluate the efficacy of IONM for preventing permanent RLN palsy and its cost-effectiveness. Nonetheless, this meta-analysis provides important data to inform clinical decision-making regarding the use of IONM in endoscopic thyroid surgery.

## Ethical approval

Not applicable.

## Consent

Not applicable.

## Sources of funding

No external funding was received for this study.

## Author contribution

I-W.C. and K-C.H. wrote the main manuscript text and W-T.W. prepared Figure 1. All authors read and approved the final version of the manuscript.

## Conflicts of interest disclosure

The authors declare no conflicts of interest.

## Research registration unique identifying number (UIN)

Not applicable.

## Guarantor

Kuo-Chuan Hung.

## Data availability statement

The datasets used and/or analyzed in the current study are available from the corresponding author upon reasonable request.

## Provenance and peer review

This paper was not invited.
